# Brain oscillation recordings of the audience in a live concert-like setting

**DOI:** 10.1007/s10339-021-01072-z

**Published:** 2021-12-27

**Authors:** Mari Tervaniemi, Saara Pousi, Maaria Seppälä, Tommi Makkonen

**Affiliations:** 1 Cicero Learning, Faculty of Educational Sciences, University of Helsinki, POB 9, 00014 University of Helsinki, Finland; 2 Cognitive Brain Research Unit, Department of Psychology and Logopedics, Faculty of Medicine, University of Helsinki, POB 21, 00014 University of Helsinki, Finland

**Keywords:** Music improvisation, Music performance, Creativity, EEG oscillation, Alpha, Theta

## Abstract

There are only a few previous EEG studies that were conducted while the audience is listening to live music. However, in laboratory settings using music recordings, EEG frequency bands theta and alpha are connected to music improvisation and creativity. Here, we measured EEG of the audience in a concert-like setting outside the laboratory and compared the theta and alpha power evoked by partly improvised versus regularly performed familiar versus unfamiliar live classical music. To this end, partly improvised and regular versions of pieces by Bach (familiar) and Melartin (unfamiliar) were performed live by a chamber trio. EEG data from left and right frontal and central regions of interest were analysed to define theta and alpha power during each performance. After the performances, the participants rated how improvised and attractive each of the performances were. They also gave their affective ratings before and after each performance. We found that theta power was enhanced during the familiar improvised Bach piece and the unfamiliar improvised Melartin piece when compared with the performance of the same piece performed in a regular manner. Alpha power was not modulated by manner of performance or by familiarity of the piece. Listeners rated partly improvised performances of a familiar Bach and unfamiliar Melartin piece as more improvisatory and innovative than the regular performances. They also indicated more joy and less sadness after listening to the unfamiliar improvised piece of Melartin and less fearful and more enthusiastic after listening to the regular version of Melartin than before listening. Thus, according to our results, it is possible to study listeners’ brain functions with EEG during live music performances outside the laboratory, with theta activity reflecting the presence of improvisation in the performances.

## Introduction

The traditional approach in studies on the neural underpinnings of music perception and cognition has emphasised firm experimental control over the stimulation and the research context. This led to studies being conducted in the laboratory environment. In particular, when auditory event-related potential (ERP) technique as a time-locked derivative of electroencephalogram (EEG) was used, highly repetitive and simplified acoustical stimulation paradigms were employed. This approach was very fruitful when the field of neurosciences of music was just emerging; pioneering findings highlighted cortical functions as the basis for musical expertise in auditory (Pantev et al. 1998; Schneider et al. 2002) and somatosensory (Elbert et al. 1995) modalities. Subsequently, subcortical structures also received well deserved attention as the first hub for neuroplasticity in audition (Wong et al. 2007).

However, when scientific interests broadened from perception and cognition to music performance and social aspects of music appreciation, the limitations of laboratory studies became clear (for reviews, see Brattico and Tervaniemi 2010; Tervaniemi 2012; D’Ausilio et al. 2015).^1^ Along with the emergence of social and affective neurosciences with naturalistic empirical settings, neurosciences of music also started to utilise methods and paradigms that are closer to real-life musical activities. Consequently, various attempts were made to record brain activity while playing a musical instrument. For EEG recordings in a solo setting, this was quite possible already in early 2000 (Kristeva et al. 2003). In investigating the performance of an ensemble, EEG recordings were conducted outside the laboratory environment only less than decade ago (Babiloni et al. 2011; 2012). In brain-mapping studies using functional magnetic resonance imaging (fMRI), this attempt made the researchers construct special musical instruments that were free of ferromagnetic objects. Currently, studies on MR- and MEG-compatible keyboards (Limb & Braun 2008; Boasen et al. 2018) and very recently also on an MR-compatible cello (Segado et al. 2018; Wollmann et al. 2018) have been successfully conducted and reported. These studies have revealed broad brain networks to be involved particularly in creative (e.g. improvisational) music performances with the highest degrees of expertise (for a review, see Loui 2018).

From the viewpoints of music psychology and music education, the interests of investigating the neural basis of music perception, creativity, and performance can also be merged. One intrinsic goal of a music performance is to evoke music emotions and interest in listeners. By investigating the psychological and neural concomitants of live music performance in the audience, we can indirectly determine how successful the performance was in accomplishing this goal. From the viewpoint of neuroscience, we might thus be able to reveal the neural underpinnings of music emotions as evoked by live music. Even if in most occasions music is listened to using recordings, live music performances are still favoured by many music lovers. The social cohesion emerging within the audience and also the contact between the musicians and the audience are highly valued, leading to higher appreciation of live than recorded music (Merrill et al. 2021). To improve the ecological validity of the empirical studies within neurosciences of music, we thus also need to develop paradigms and methodologies to record brain activity outside the laboratory during live performances.

As the pioneers of this initiative, Dolan et al. (2013, 2018) conducted EEG recordings during live music performances in a concert-like setting using four music listeners and three musicians as their participants. They found that in all musicians and in three out of four listeners, the EEG signal complexity (as quantified using Lempel–Ziv analyses; for details, see Dolan et al. 2018) was higher during improvised than during regular music performance. This was taken to indicate increased awareness and alertness during the improvised performances than during the regular performances.

The main goal of the current contribution was to determine whether brain electric oscillatory activity of the listeners would differ between live music performances when the musical pieces were performed according to the musical score or when they included improvisational features and were more freely performed. We also compared performances of familiar (Arioso by Bach) and unfamiliar compositions (piece by a Finnish national composer, Melartin). Of particular interest were alpha (8–13 Hz) and theta (4–7 Hz) frequency bands, which both have been identified to reflect creative processes also in musically relevant laboratory studies using EEG and magnetoencephalographic recordings (e.g. Jäncke et al. 2015; Markovic et al. 2017; Lopata et al. 2017; Boasen et al. 2018). More specifically, alpha oscillations have been found to reflect the vigilance of the listener (Jäncke et al. 2015) and theta oscillations to reflect the internal music-driven mental state (mind wandering) of the listeners (Markovic et al. 2017; Jäncke et al. 2015).

To mimic an authentic concert experience, the recordings were conducted in parallel with four listeners who were listening to a live performance of chamber music. Based on previous laboratory-based findings introduced above, our hypothesis was that alpha activity would be suppressed, and theta activity enhanced when the performance included improvisational features and, further, that these effects would be stronger when the audience was familiar with the musical piece and previously acquired mental schemata guide the perceptual processes. To this end, a chamber music trio prepared two versions of a piece composed by Bach (a familiar piece that was performed by the live trio with and without improvisatory elements) and by a national composer Melartin (unfamiliar piece that was performed by the live trio with and without improvisatory elements).

## Materials and methods

### Participants

The EEG and questionnaire data result from 16 participants (20–33 years, mean age 25.9 years, SD 3.92; 10 females). All participants reported to be right-handed, free of neurological diseases and medication, and to have normal hearing. They all also reported a long background in music activities: They had all played a musical instrument for 10–28 years (mean 17.4), and 12 of them had obtained professional studies in music. When asking for their preference for classical music (scale 1–7), their average preference score was 6.0 (SD 1.1).

### Procedure

The study was conducted in a total of four sessions during 2 days in the seminar rooms of the University of Arts, Helsinki (eight participants) and of the University of Helsinki (eight participants). The EEG of four participants was simultaneously recorded while listening to the live performance of a chamber trio (see Sect. “Music performance” below). The seminar rooms were spacious with removable furniture, had adequate lighting, and had no noise originating from the neighbouring rooms, corridors, or streets.

The participants were seated in front of the trio at about a 3-m distance. They were instructed to take a comfortable position and sit as steadily as possible. They were told that the trio will play four different musical pieces and that the study was about different practices in music performances.

In the same occasion, the participants also completed the background information questionnaire and questionnaires about their emotional state and their views about the performances (see Sect. “Questionnaires” below).

The experimental protocol was conducted in accordance with the Declaration of Helsinki and approved by the University of Helsinki Ethical review board in the humanities and social and behavioural sciences. All participants provided written informed consent.

In total, the experimental session took 2–3 h, depending on the time needed for completing the questionnaires and the EEG cap attachment and removal. The EEG recording, including breaks during which the participants completed part of the questionnaires, took about half an hour. The participants were compensated for their time with vouchers valid for culture and sport activities. The musicians were given monetary compensation.

### Music performance

The chamber trio consisted of the same musicians who performed the pieces altogether four times. The trio consisted of violin, cello, and kantele (traditional Finnish string instrument that belongs to the family of zithers). The musicians forming the trio had been rehearsing joint improvisation and had received training in classical improvisation. They played two versions of J.S. Bach’s Arioso from Cantata BWV 156 – Adagio and Melartin’s Berceuse, Op. 83 No 7, both arranged for this trio.^2^ One version was played based on the musical score and the conventions of classical music performances while another version was played using an improvisational style of classical music. Here, one musician elaborated their part while the others played according to the score. All musicians, one by one, played in an improvisational style.

The order of the performances was as follows: during the first recording day (eight participants), regular Bach, improvised Bach, improvised Melartin, regular Melartin; during the second recording day (eight participants), regular Melartin, improvised Melartin, improvised Bach, regular Bach. Before the study was conducted, all participants were instructed to listen to Bach’s Arioso at home to ensure familiarity. They were given no information about the other musical piece (Melartin) before the end of the study. Additionally, EEG of the participants was recorded for 30 s to establish baseline values before the first performance. For practical reasons, baseline was only recorded before the first performance. Duration of the shortest music performance was 80 s.

### Questionnaires

In the context of the recordings, the participants completed a background information questionnaire and the Short Five personality inventory. The Short Five data will be reported elsewhere.

Additionally, after each performance, the participants completed a questionnaire on musical aspects of the performance (improvisatory in character, innovative in approach, emotionally engaging, musically convincing, risk-taking, and being interesting) using a scale from 1 (least) to 5 (most). The participants also completed an emotional state questionnaire before and after each performance. Here they were asked to indicate how strongly they felt basic emotions (joy, sadness, anger, fear, disgust, enthusiasm). The scale was also between 1 (least) and 5 (most) in this questionnaire.

### EEG recordings and analyses

Continuous EEG data were collected with BrainVision LiveAmp amplifiers (Brain Products GmbH, Germany) and BrainVision Recorder software version 1.21.0004. Using the 10/20 system, 32 BrainVision actiCAP electrodes were attached to the scalp of the participants with an Easycap electro cap (EASYCAP GmbH, Germany) and Signa gel (Parkers Laboratories, Inc., USA). The ground electrode was positioned at Fpz.

EEG was sampled at 500 Hz (bandwidth DC–131 Hz). The recording was started before the trio performance began and stopped after the performance had ended. The time points of the performance onset/offset were manually annotated to the continuous EEG files during the recording.

After visual inspection of the EEG files, the data were preprocessed using EEGLAB software version 2019.1 (Delorme andMakeig 2004) and an in-house toolbox (CBRUplugin, implemented by the fourth author) running in MATLAB R2019a (The Mathworks Inc., USA). Continuous data were re-referenced to Cz and band-pass filtered (FIR 1–45 Hz; 6 dB cut-off frequencies 0.5 Hz and 47.8 Hz, respectively). Data were segmented to contain the first 80 s of listening conditions and 30 s for the baseline. For practical reasons, baseline was only recorded before the first performance.^3^

Thereafter, the data were visually inspected for artefactual channels. ICA decomposition (runica) was run excluding the artefactual channels (mean 1.25, max 6, min 0 per participant) and used for removing artefacts caused by eye movements. All components labelled as eye artefacts (having a typical topography of either a blink or horizontal movement) with likelihood higher than 94% were removed (1.81 components per participant on average). Labelling was done using ICLabel (Pion-Tonachini et al. 2017). After this, bad channels required by the analysis were interpolated (three participants with 1, 2, and 3 channels, respectively).

*Power Spectral density* (PSD) was calculated over an 80-s window (which was the duration of the shortest performance) during a given music piece and over a 30-s window during rest by using the spectopo function of EEGLAB. FFT window was set to 2048 samples with 50% overlap. This resulted in a frequency resolution of 0.24 Hz. The mean PSD was calculated in the Theta (4–7 Hz) and Alpha (8–13 Hz) bands for all participants separately in each condition. The data were averaged further for the analysis of ROIs, which were left frontal (F3, F7, FC1, FC5), right frontal (F4, F8, FC2, FC6), left central (C3, T7, CP1, CP5), and right central (C4, T8, CP2, CP6). Four regions were used to keep the statistical analysis rather simple and to focus on the areas of scalp that display the most prominent EEG oscillatory effects (e.g. Mikutta et al. 2014). While posterior channels would also have been valuable for the analyses of alpha band, due to poor electrode–skin contact in many posterior and occipital channels (resulting in poor signal-to-noise quality of the EEG), we could only find two subsets of four electrodes with sufficient signal quality. Individual baseline values were subtracted from each ROI mean value within each participant.

## Results

### EEG oscillations: theta

**Fig. 1:**
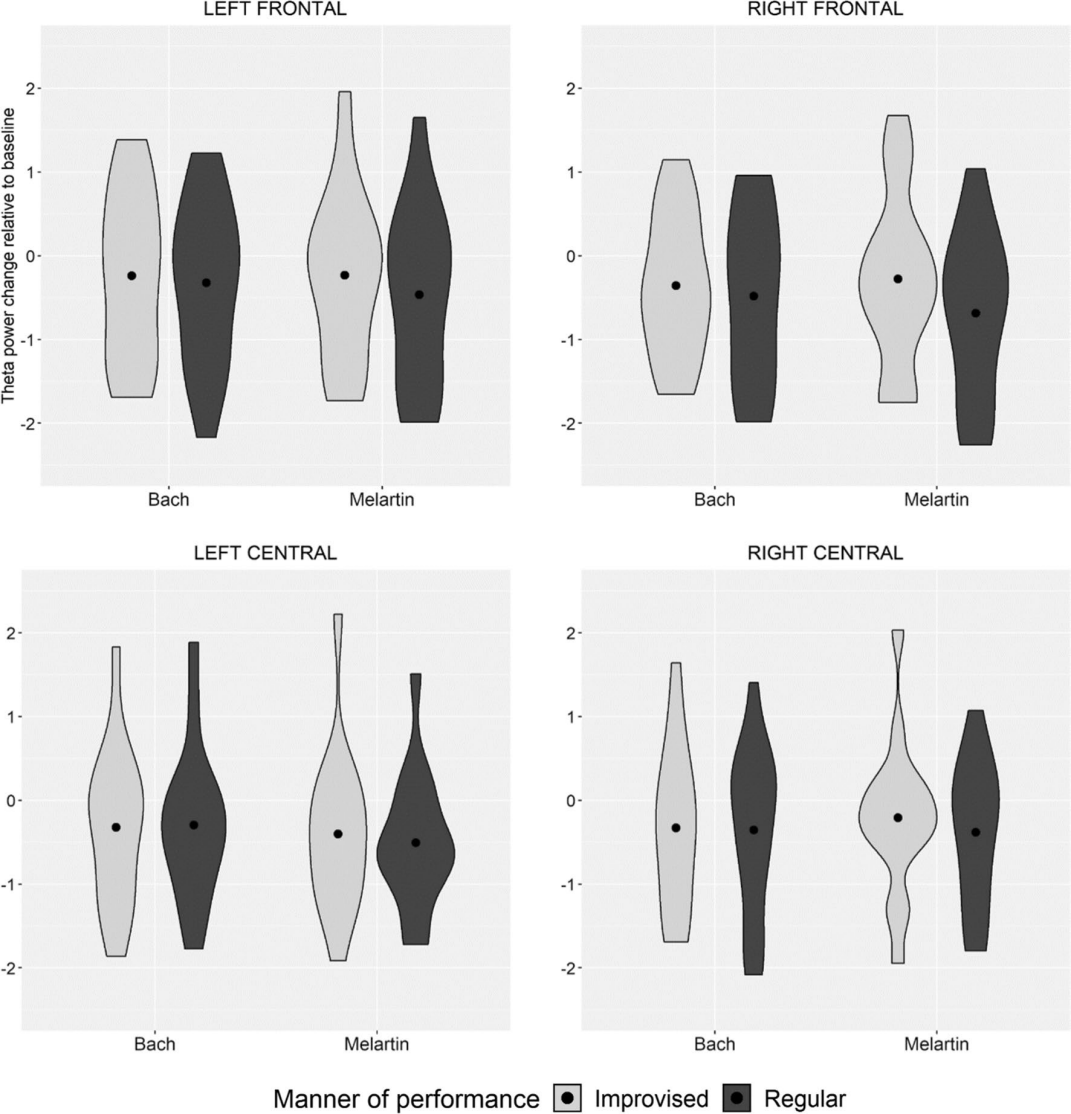
Condition-wise power spectrum density mean values measured at four regions of interest (left frontal, right frontal, left central, and right central) for improvised (light gray) and regular (dark gray) performances. Note that the PSD mean values describe the change of the power spectrum density compared to the baseline condition (listening minus baseline)

The theta activity was higher for improvised than for regular performances (Tables 1 and 2; Fig. 1). This was indicated by the main effect of manner of performance when compared across all four ROIs using a 3-way repeated measures ANOVA with factors ROI (left frontal, right frontal, left central, right central) × manner of performance (improvised, regular) × familiarity (familiar, unfamiliar); *F*(1, 15) = 7.60, *p* = 0.015). There was no main effect of the familiarity of the pieces or statistically significant interactions.

**Table 1:** Means and Standard Deviations of Alpha and Theta Power (averaged across all four ROIs)

	Alpha		Theta	
	Mean	SD	Mean	SD
Regular Melartin	− 0.40	0.89	− 0.51	0.88
Regular Bach	− 0.37	0.77	− 0.36	0.93
Improvised Melartin	− 0.10	0.71	− 0.28	0.94
Improvised Bach	− 0.30	0.67	− 0.31	0.89

**Table 2:** *p* values in Three-way Repeated Measures ANOVA: Theta

	df	MS	*F*	*P*
Manner of performance (Regular vs. Improvised)	1, 15	1.24	7.60	0.015
Piece (Familiar Bach vs. Unfamiliar Melartin)	1, 15	0.21	0.52	0.48
ROI	3, 45	0.27	1.03	0.39
Manner of performance (Regular vs. Improvised) × Piece (Familiar Bach vs. Unfamiliar Melartin)	1, 15	0.51	2.93	0.11
Manner of performance (Regular vs. Improvised) × ROI^a^	3, 45	0.22	2.81	0.07
Piece (Familiar Bach vs. Unfamiliar Melartin) × ROI	3, 45	0.10	2.73	0.06
Manner of performance (Regular vs. Improvised) × Piece (Familiar Bach vs. Unfamiliar Melartin) × ROI	3, 45	0.02	0.41	0.74

^a^Greenhouse–Geisser correction has been used but the original degrees of freedom are reported

### EEG oscillations: alpha

There were no statistically significant effects according to manner of performance or familiarity of the musical piece (Tables 1 and 3; Fig. 1). This was indicated by the lack of main effects or interactions when comparing the alpha activity across all four ROIs using a 3-way repeated measures ANOVA with factors ROI (left frontal, right frontal, left central, right central) x manner of performance (improvised, regular) x familiarity (familiar/unfamiliar piece); all *p* values between 0.11 and 0.76) (Table 3).

**Table 3:** *p* values in Three-way Repeated Measures ANOVA: Alpha

	df	MS	*F*	*p*
Manner of performance	1, 15	2.33	2.86	0.11
Piece (Familiar Bach vs. Unfamiliar Melartin)	1, 15	0.48	0.30	0.59
ROI	3, 45	1.35	1.36	0.27
Manner of performance × Piece (Familiar Bach vs. Unfamiliar Melartin)	1, 15	0.85	1.29	0.27
Manner of performance × ROI^a^	1, 15	0.04	0.22	0.76
Piece (Familiar Bach vs. Unfamiliar Melartin) × ROI	3, 45	0.10	1.17	0.33
Manner of performance x Piece (Familiar Bach vs. Unfamiliar Melartin × ROI	3, 45	0.09	1.40	0.26

^a^Greenhouse–Geisser correction has been used but the original degrees of freedom are reported

### Questionnaire data

After each performance, the participants rated their impressions about the pieces’ attributes, such as improvisatory in character, innovative in approach, emotionally engaging, musically convincing, risk-taking, and being interesting using a 5-step Likert scale from 1 (least) to 5 (most). By these descriptors, we sought to determine the perceived musical attributes of the performances. Ratings between regular and improvised performances were compared using non-parametric Wilcoxon tests, as the data were not normally distributed. For descriptive data, see Table 4 and statistical analyses for each attribute below.

**Table 4:** Average scores of ratings along six musical features on a scale of 1 (least) to 5 (most)

		Mean	SD
Improvisatory	Bach regular	1.19	0.40
	Bach improvised	3.44	1.21
	Melartin regular	1.56	0.89
	Melartin improvised	2.06	0.85
Innovative	Bach regular	1.94	0.77
	Bach improvised	3.19	1.11
	Melartin regular	2.33	0.98
	Melartin improvised	2.86	0.89
Musical	Bach regular	4.31	0.70
	Bach improvised	3.75	0.86
	Melartin regular	4.06	0.68
	Melartin improvised	3.94	0.57
Risk-taking	Bach regular	2.86	1.02
	Bach improvised	3.31	0.95
	Melartin regular	2.75	1.00
	Melartin improvised	3.06	0.85
Emotional	Bach regular	3.63	1.09
	Bach improvised	3.31	1.08
	Melartin regular	3.38	0.81
	Melartin improvised	3.75	0.68
Interesting	Bach regular	3.44	0.96
	Bach improvised	3.63	0.81
	Melartin regular	3.25	1.00
	Melartin improvised	3.56	1.03

#### Improvisatory

The audience rated the improvised versions as more improvisatory than the regular versions (Bach *Z* = − 3.24, *p* = 0.001, Melartin *Z* = − 2.31, *p* = 0.021).

#### Innovative

The audience rated the improvised versions as more innovative than the regular versions (Bach *Z* = − 3.01, *p* = 0.003, Melartin *Z* = − 2.11, *p* = 0.035).

#### Musical

The regular version of Bach was rated more musical than its improvised version (*Z* = − 2.31, *p* = 0.021).

#### Risk-taking

There was a tendency for the improvised version of Melartin to be rated as more risk-taking than the regular version (*Z* = − 1.89, *p* = 0.059).

#### Emotional

There were no significant differences in terms of ratings for the degree of emotionality.

#### Interesting

There were no significant differences in terms of ratings for the degree of interest.

#### Basic emotions

The participants were also asked to complete an emotional state questionnaire before and after each performance. Here they were to indicate how strongly they felt basic emotions (joy, sadness, anger, fear, disgust, hype) by using 5-step Likert scale of 1 (least) to 5 (most). By these ratings, we aimed at determining whether music listening modulated their ratings and, if so, whether modulation was different by regular versus improvised versions. Emotional ratings given before and after listening were compared using non-parametric Wilcoxon tests since the data were not normally distributed.

Listening to Bach either as the regular or improvisatory version did not significantly modulate the emotional ratings of the listeners. After listening to improvised performance of Melartin, they reported feeling more joyful (*Z* = − 2.31, *p* = 0.021) and less sad than before listening (*Z* = − 2.12, *p* = 0.034). After listening to the regular performance of Melartin, they reported feeling less fearful (*Z* = − 2.43, *p* = 0.015) and more enthusiastic than before listening (*Z* = 2.11, *p* = 0.035).

## Discussion and conclusions

The main goal of the current contribution was to determine, along the lines launched by Dolan et al. (2013, 2018 with a total of 12 participants in these two studies), whether brain electric oscillatory activity differs between live music performances when the musical pieces were performed according to the musical score or when they included improvisational elements and were more freely performed. Our recordings were conducted in a concert-like out-of-the-laboratory setting in four subsequent recordings with a total of 16 participants. The hypothesis was that alpha and theta activity would be modulated when the performance included improvisational features and, further, that this modulation would be stronger when the audience was familiar with the musical piece. We were also interested in determining whether the listeners would perceptually differentiate regular versus improvised performances and whether these performances would affect their emotional ratings.

We found that theta activity was elevated by improvised performances of Bach and Melartin pieces without any significant difference between these two. This modulation of theta activity was also reflected in perceptual ratings of the pieces; the listeners considered improvised versions as more improvisatory and innovative than the regularly performed pieces. Furthermore, they rated the regular Bach piece as more musical than the improvised Bach piece. In parallel, the emotional ratings of the listeners reflected the familiarity of the pieces; the listeners felt more joyful and less sad after the improvised version of Melartin’s piece than before it. They also reported being less fearful and more enthusiastic after listening to the regular version of Melartin’s piece than before the listening.

In contrast to our expectations, alpha activity was not suppressed during improvised performances when compared with regular performances. Such an effect, previously seen in laboratory music listening experiments when listening is compared to a resting baseline (e.g. Jäncke et al. 2015), would reflect decrease in vigilance and increase of relaxation (psychological concomitants of alpha increase). It is possible that a relatively short live performance by the chamber trio was not calming and relaxing even if it was performed in a traditional score-based manner. Alternatively, listening to live performance is more engaging as such than listening to recorded music. This might be reflected by the emotion ratings, which did not indicate any difference between regular and improvised performances of Bach. For the unfamiliar piece of Melartin, both a regular and improvised performance modulated the emotional scores as discussed above (Fig. 1).

Our evidence indicates that changes in the neuroaffective status of the listeners can be probed by theta activity and self-reports when listening to live music during EEG recordings outside a laboratory setting. Furthermore, the effects of listening on emotional ratings were more elaborated when participants were listening to an unfamiliar piece by Melartin than to familiar piece by Bach. This suggests that, at least with the current audience consisting mainly of musically trained listeners, emotional states (such as joy, sadness, fear, and enthusiasm) were best conveyed by Melartin’s piece from the romantic era, while the improvisatory characteristics and innovativeness were observed in both pieces. However, these behavioural and EEG outcomes were complementary and not closely linked.

The current contribution is consistent with prior published evidence on the sensitivity of theta oscillations to reflect the internal music-driven mental state of the listeners, which is also suggested to correspond with mind wandering (Markovic et al. 2017; Jäncke et al. 2015). However, to our knowledge, this contribution is the first to report EEG data that have been collected outside the laboratory environment with a relatively large sample of participants in the audience who provided their questionnaire and EEG data for the use of researchers.

The current contribution has interesting discrepancies regarding alpha activity. There were such discrepancies between previous studies conducted in the laboratory and the current study in a live concert setting and between emotional ratings and alpha activity. These discrepancies will drive the establishment of multimethodological studies in this field in the future. Ideally, these studies should compare behavioural and physiological measures in the laboratory and concert settings with longer music excerpts along with parametric manipulation of the familiarity of the excerpts. Furthermore, these studies should also use a more advanced procedure in randomizing the order of the musical pieces. Alternatively, such studies should have access to, for instance, 20 EEG systems at once so that all data are collected in one recording in the same order of the pieces for all participants. Even if such an arrangement would exclude the possibility to reveal any effects of the listening order, it would make the listening context more to resemble a concert setting, particularly if the audience is larger with EEG recordings being conducted for a subsample of it (for such an arrangement, see Wan et al. 2014).

We lack more elaborate analyses of the neural generators of the theta activity results observed. However, this was beyond the capabilities of our mobile 32-channel EEG recording systems. Likewise, audio analysis of the recorded music performances has not yet been performed to determine how much the four performances (Bach regular/improvised; Melartin regular/improvised) differed from each other in acoustic terms. This should be conducted in future studies by e.g. using MIR toolbox (Lartillot et al. 2008), which enables quantification of over 100 acoustic and music-structural features.

To conclude, we consider the current contribution to open new avenues for research on musical creativity and music emotion induction by combining perspectives of music psychology and the neurosciences of music in an ecologically valid context.

## Data Availability

The datasets collected during the current study are not publicly available as the participants were not informed of the possibility that their data would be openly available. We promised to keep the data anonymous and restricted to the researchers of this project.

## Footnotes

1 It is of note that during past decade, development of relevant empirical paradigms and analyses methods have enabled ERP technique to be useful also in naturalistic music paradigms, see, e.g. Vuust et al. 2011, Tervaniemi et al. 2014, Poikonen et al. 2016, and Di Liberto et al. 2020.

2 Erkki Melartin (1875–1937) was a Finnish composer whose broad repertoire represents late romantism with some influences from impressionism, expressionism, and folk music.

3 However, we also tested whether the result is dependent on the selected baseline period. Some artifactual data had to be rejected for this re-analysis (outlier values of one participant in the improvised Bach condition were more than 10  × group standard deviation in each ROI). The main finding of the paper was replicated, theta activity being significantly higher (*p* = 0.034) for improvised performances compared to the regular performance.
